# Employers’ views on the promotion of workplace health and wellbeing: a qualitative study

**DOI:** 10.1186/s12889-015-2029-2

**Published:** 2015-07-11

**Authors:** Melanie Pescud, Renee Teal, Trevor Shilton, Terry Slevin, Melissa Ledger, Pippa Waterworth, Michael Rosenberg

**Affiliations:** Health Promotion Evaluation Unit, 35 Stirling Highway, Crawley, 6009 Australia; Regulatory Institutions Network (RegNet), The Australian National University, Acton, Australia; National Heart Foundation (WA), Subiaco, Australia; Cancer Council WA, Shenton Park, Australia

**Keywords:** Workplace health promotion, Employers, Focus groups, Health and Wellbeing

## Abstract

**Background:**

The evidence surrounding the value of workplace health promotion in positively influencing employees’ health and wellbeing via changes to their health behaviours is growing. The aim of the study was to explore employers’ views on the promotion of workplace health and wellbeing and the factors affecting these views.

**Methods:**

Using a qualitative phenomenological approach, 10 focus groups were conducted with employers selected from a range of industries and geographical locations within Western Australia. The total sample size was 79.

**Results:**

Three factors were identified: employers’ conceptualization of workplace health and wellbeing; employers’ descriptions of (un)healthy workers and perceptions surrounding the importance of healthy workers; and employers’ beliefs around the role the workplace should play in influencing health.

**Conclusions:**

Progress may be viable in promoting health and wellbeing if a multifaceted approach is employed taking into account the complex factors influencing employers’ views. This could include an education campaign providing information about what constitutes health and wellbeing beyond the scope of occupational health and safety paradigms along with information on the benefits of workplace health and wellbeing aligned with perceptions relating to healthy and unhealthy workers.

## Background

Prevention of chronic diseases is an important global health issue [1, 2]. Unhealthy lifestyle practices such as poor nutrition, physical inactivity, alcohol use, and smoking can contribute to the chronic disease burden and significantly impact population health outcomes [3, 4]. At the workplace, unhealthy lifestyles have been shown to reduce productivity and increase absenteeism and presenteeism [3, 5]. Workplaces provide access to a considerable proportion of the adult population and as such are an ideal setting for health promotion initiatives [3, 6–8]. Therefore, programs aimed at the workplace have the potential to reach a large segment of the population who might not be exposed to other health promotion initiatives [9]. Workplaces also provide an opportunity for tailoring programs and health messages to meet specific needs of industry segments and demographic groups.

Health promotion efforts are usually directed towards improving the health of a workforce through initiatives such as health risk assessments, vaccinations, and wellness activities targeted at improving healthy eating, physical activity, cigarette use, alcohol consumption, and mental health outcomes [5, 6, 10, 11]. Another area that influences the health of employees, but is often considered separate from health promotion, is occupational health and safety or health protection. Occupational health and safety encompasses efforts that prevent injury or illness due to workplace specific exposures, by conducting safety training, environmental modification, and the provision of and use of personal protective equipment [6]. There is often an overlap between health promotion and health protection (e.g., creating smoke free zones) and there are calls to integrate both areas because both contribute to the overall health and wellbeing of employees [6]. Further, in many jurisdictions, health protection is codified in occupational health and safety or similar regulations. Health and wellbeing in the workplace can be viewed as a broad concept comprised of personal satisfaction; work-life satisfaction; and general health which is a combination of mental/psychological health and physical/physiological health [12].

The evidence surrounding the value of workplace health promotion in positively influencing employees’ health and wellbeing via changes to their health behaviours is growing [13–19]. Several factors influencing the implementation of workplace health promotion programs have been identified [20]. These factors act at multiple levels of influence with implementation determinants including characteristics of the socio-political context, the organisation, the implementer, the intervention program, and the individual [20]. A range of stakeholders share an interest in workplace health promotion ranging from employers and employees to insurance companies, occupational physicians, various government departments, labour unions, universities, and organisations with a health promoting focus [21]. At the level of the organisation, two under-researched aspects of particular interest are employers’ perceptions regarding the importance of having healthy workers and the responsibility these staff feel towards influencing worker health [21–24]. Their perceptions are important because an integral part of implementing health promoting policies and programs into the workplace is obtaining support from those in managerial or leadership roles [6, 25–29].

Literature exploring the perceived role of the workplace in influencing employees’ health behaviours is limited, with much of the work carried out solely exploring employees’ views (e.g., Pridgeon and Whitehead [30]), and only a few studies investigating the views of managers or employees in leadership roles (e.g., Linnan et al. [22]; Hannon et al. [31]; Audrey and Procter [24]). In addition, limited research appears to have been conducted in an Australian setting. Qualitative and quantitative research carried out with stakeholders from various organisations in the United States has revealed that there is perceived merit in workplace health programs because they were able to improve employee morale, reduce health care costs, increase productivity, reduce absenteeism, and contribute to the positive promotion of the company image [22, 31]. Several barriers to their implementation have, however, been cited including costs, time scarcity, logistical issues, and cultural barriers [22, 31].

The literature indicates that creating a healthy workplace is important [13–17] and given that many workplaces do implement health promoting programs for their workers, this would suggest that some employers feel they have a role to play in creating healthy work environments. The difficulty is determining which aspects contributing to employees’ health those in management or leadership roles should have responsibility for and to what extent. Responsibility regarding occupational health and safety is of clear importance, and often legislated, while the lines are somewhat blurred and discretionary in relation to activities covered under the broader topic of health and wellbeing [6]. While employers in Linnan et al.’s study [22] could see the merit in implementing health and wellbeing programs, many did not believe it was their responsibility to make such programs available; ensuring worker safety was, however, perceived to be a key responsibility. More research needs to be conducted exploring employer perceptions relating to responsibility regarding health and wellbeing at work.

Explanations of healthy workplaces are present in the literature. For example, Sauter et al. [32] define a healthy workplace as one that “maximizes the integration of worker goals for well-being and company objectives for profitability and productivity” (p. 250). There is, however, a lack of information on employer perceptions surrounding what defines a healthy worker and, conversely, what defines an unhealthy worker. Exploring their perceptions may assist with understanding how such perceptions tie into views relating to responsibility for worker health. The present study used a qualitative approach to explore some of the factors affecting employers’ views on the promotion of health and wellbeing in the workplace. Qualitative methods were deemed ideal given the paucity of research in this area and the necessity to make meaning regarding employers’ definitions and perceptions.

## Methods

Phenomenology was used to underpin the research process. This methodology promotes an examination of the lived experiences of individuals and draws on tenets from a number of disciplines including psychology, education, and philosophy [33]. A qualitative cross-sectional design involving focus groups was utilised to gather information about employers’ views on workplace health and wellbeing and the factors affecting these views. The study protocol was approved by the University of Western Australia Human Research Ethics Committee.

### Setting and sample

A purposive sample of participants was obtained. All participants were 25 years of age or older and were business owners or managers or were responsible for organisational occupational health and safety, and so in a position to influence changes to the workplace in terms of health and wellbeing (i.e., a health and safety officer, team manager or leader, supervisor, or trainer). For the purposes of this study they are referred to as employers because they were acting as workplace representatives. To elicit a broad range of responses and identify relevant issues for various business groups, participants were men and women, selected from a range of geographical locations and industries; which were defined as blue or white collar.

A social research agency was employed to recruit the focus group participants via random digit dialling. As part of the recruitment process, potential participants were informed that they were being recruited to join a discussion on health and wellbeing in the workplace. All participants provided their informed written consent to take part in the study prior to the commencement of data collection. Participants were offered AUD$80 (USD$65) reimbursement for their time and expenses.

### Focus groups

A total of 10 focus groups were conducted to collect the data. Ten participants were invited into each group, with attendance rates ranging from four to 10. The total sample size was 79. The demographic profiles of the groups are presented in Table 1.

**Table 1:** Demographic profile of groups (n = 79)

Group	Location	Industry	Men	Women	Participants
1	Rural	Blue collar	7	2	9
2	Rural	White collar	0	6	6
3	Rural	Blue collar	5	3	8
4	Rural	White collar	8	2	10
5	Metropolitan	Blue collar	4	5	9
6	Metropolitan	Blue collar	0	8	8
7	Metropolitan	Blue collar	4	0	4
8	Metropolitan	White collar	3	6	9
9	Metropolitan	White collar	0	8	8
10	Metropolitan	White collar	8	0	8
Total			39	40	79

Focus groups were chosen as the data collection method because (1) the area under investigation was non-sensitive and topical (2) they enabled a diverse range of opinions to be accessed, (3) they provided a space to explore group dynamics and traits (both between and within groups), and (4) they were logistically appropriate. The focus groups were conducted using a semi-structured approach, as per Fontana and Frey [34]. The lead author facilitated each of the groups and a research assistant was also present for the purpose of taking field notes. The discussion topics were introduced in each group in a similar order, with some variations occurring according to the spontaneous mention of particular issues by the participants. The discussion began with a series of word associations relating to healthy workers; unhealthy workers; and workplace health and wellbeing. Participants were asked to write down on a piece of paper, “What springs to mind when you hear the words ‘healthy worker’?” The same activity was repeated changing the words to ‘unhealthy worker’ and then ‘workplace health and wellbeing’. Each participant read out their list of words or phrases to the group. These conversations evolved into discussions around the perceived importance of healthy workers, the role of the workplace in influencing health and wellbeing, and implementation of health and wellbeing initiatives in workplaces. Overall, the participants were very engaged in the topics being discussed. The groups ran from 61 to 89 min. Each group was audio recorded and recordings were subsequently transcribed verbatim and de-identified to ensure the confidentiality of participants.

### Data analysis

All transcripts (including field notes) were uploaded into QSR NVivo10 (QSR International Pty Ltd, Australia) for coding and analysis. Analysis commenced prior to coding as the lead researcher engaged the research assistant present at the focus groups in an analytical conversation at the end of each group as a strategy for identifying and unpacking concepts as they unfolded. In particular, participant comments conveying significant emotion were noted to further assist in the examination of meaning. Subsequently, a coding hierarchy was created using the discussion guide and themes emerging from the data [35]. The initial coding framework was approved by the members of the research team.

To begin with, the data were coded line by line to assist with identification of concepts which were subsequently grouped together to form categories [36]. Changes were made to the categories throughout the coding and analysis process as relationships between concepts became clear; this involved both grouping and modification of categories in conjunction with an ongoing dialogue with the research team. While all of the data were coded, data saturation was reached prior to the end of coding; at this point it was apparent that no new information was emerging from the data and repetition of concepts became consistent. Once coding was complete, matrix searches were used to facilitate identification of issues most salient to different groups of participants based on characteristics such as geographic location, industry types, business size, and gender.

A number of steps were undertaken to ensure the trustworthiness of the research. These included (1) triangulation via the inclusion of a range of participants in various roles residing in different locations and working in variable sized businesses, (2) peer review via ongoing discussion with the research team as well as colleagues not working on the project on data coding and analysis processes, and (3) an audit trail involving the assistance of members of the research examining the coding and analysis processes and interpretation of findings [37].

## Results

**Fig. 1:**
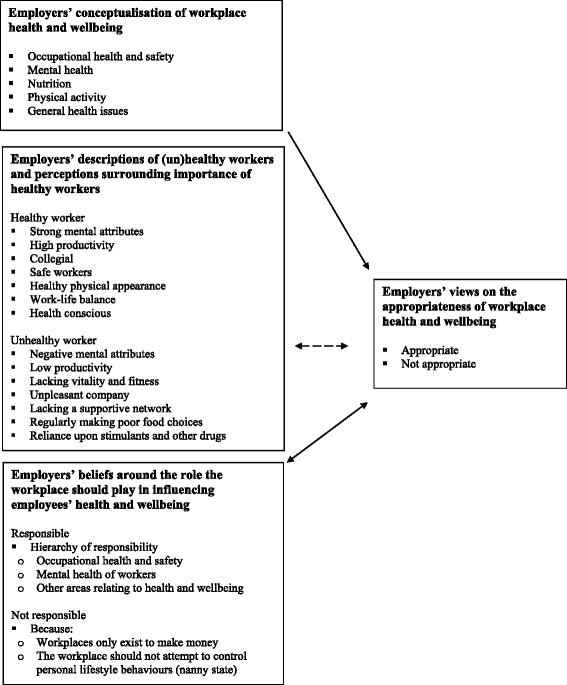
Employers’ views on the appropriateness of workplace health and wellbeing

Three main factors were identified as influencing employers’ views on the promotion of health and wellbeing in the workplace and these are summarised in Fig. 1. These included (1) employers’ conceptualisation of workplace health and wellbeing, (2) employers’ descriptions of (un)healthy workers and perceptions surrounding importance of healthy workers, and (3) employers’ beliefs around the role the workplace should play in influencing employee’s health and wellbeing.

### Employers’ conceptualisation of workplace health and wellbeing

Previous research has shown that there is often confusion regarding what workplace health and wellbeing entails [12]. Therefore, to place conversations on the topic of workplace health and wellbeing in context, it was necessary to understand participants’ perceptions of what it means. By carrying out the word association activity in the focus groups, it enabled participants to think broadly on the topic, which resulted in a wide range of responses. Workplace health and wellbeing was perceived to encapsulate one or all of the following areas: occupational health and safety, mental health, nutrition, physical activity, and general health issues. Responses varied from group to group and within groups.

The rural groups viewed health and wellbeing in the workplace as mostly relating to occupational health and safety. This was particularly the case in the two focus groups that were set in a large mining town where safety was a prominent issue. Because of the narrow focus on occupational health and safety in these two groups, many conversations pertaining to other aspects of workplace health and wellbeing (e.g., how lifestyle health behaviour risk factors can be reduced) were somewhat diminished compared to the other groups. These participants were focused on safety issues relating to the avoidance of accidents and injuries. The metropolitan groups, especially those comprised of white collar workers, were more likely to view health and wellbeing in the workplace as encompassing a broader range of issues touching upon all themes identified. Women, more so than men, focused on health in a more holistic manner. Across all groups, a particular emphasis was placed on mental health issues, mostly in relation to work-related and personal stress.

*“Work/life balance, mental health, and then stress…I’m dealing with a lot of people with mental health and stress issues at the moment.”* (FG9, metropolitan, white collar, women)

### Employers’ descriptions of (un)healthy workers and perceptions surrounding importance of healthy workers

To effectively investigate the factors influencing employers’ views on health and wellbeing in the workplace, it was important to understand their perceptions surrounding what defines a healthy worker and, conversely, what defines an unhealthy worker. Several terms were used to describe both the healthy worker and the unhealthy worker and these were subsequently grouped into themes. No weighting was given to any one aspect over another when depicting the workers; however the frequency of comments provided indicated that the state of workers’ mental health was the most salient defining feature.

The healthy worker was described in terms of positive mental attributes. Examples of these attributes included being alert, cheerful, focused, confident, and calm. Healthy workers could also be recognised by their high productivity, their collegiality, their use of safe work practices, their healthy physical appearance, their ability to maintain a work-life balance, and their health consciousness (e.g., maintaining a healthy diet and sleeping enough). In contrast, unhealthy workers were described as having negative mental attributes (e.g., being stressed, having a negative attitude, lacking self-respect, and visibly unhappy), experiencing significantly reduced productivity, lacking vitality and fitness, being unpleasant company, not having a supportive network, regularly making poor food choices, and being reliant on stimulants (e.g., caffeine or cigarettes) and other drugs.

Most participants reported that healthy workers were of utmost importance in the workplace. Reasons provided for this perception included the increased productivity, more positive attitudes, and greater levels of safety perceived to be associated with healthier workers. A link, however, between the importance of healthy workers and a need to introduce health and wellbeing initiatives at work was not often made by participants, hence the dashed line instead of continuous arrow presented in Fig. 1.

Productivity was the most frequently mentioned outcome of importance in relation to having healthy workers. Greater productivity was believed to translate into higher business profitability. Unhealthy workers were perceived to be more likely to take sick days or carry out their duties in a slow manner due to fatigue, thus costing the business. It was also noted that there were difficulties associated with the need to fulfil duties when workers with unique roles were absent.

*“It’s very important because it affects the running of the business. If you’ve got numerous people with sick days and that sort of thing, it has a financial impact on the business.”* (FG10, metropolitan, white collar, men)

*“I think it’s important because our employees are actually friends and they’re people we care about and we want them to be well and healthy for their own sake. When somebody is not well their production is down and that costs you as a small business, but the money side is less important than the moral side of their wellbeing.”* (FG1, rural, blue collar, men and women)

Employee attitude and demeanour were also highlighted as typical traits of a healthy worker that were highly desirable in the workplace setting. For example, interactions between fellow employees and customers or clients could be positively or negatively influenced depending on the mood of staff members. The morale of the team was listed as particularly important.

*“If someone is happy and cheerful, well they’re healthy, they give better customer service, they work better with their colleagues, and you’ve got a happier team. If they’re cheerful they will joke amongst each other. Having been in a position of being an unwell boss, I’ve known how grumpy and glum and cranky and irritable I can be.”* (FG2, rural, white collar, women)

*“They want to be there, I mean obviously they boost your morale, I mean the morale is going to be up. When morale’s down everyone’s whinging, no one wants to be there, it’s toxic.”* (FG4, rural, white collar, men and women)

Healthy workers were viewed as necessary due to their role in maintaining workplace safety. This was most commonly reported in the blue collar and rural groups. Mental alertness was reported to be an integral aspect of the healthy worker; this trait was singled out as a vital feature of workers.

*“You have to have a healthy (workforce), well from where I come from, it’s the risk you know, if you’re not healthy and fit you’re a danger. You know the size of the stuff that I’m involved in someone can be killed, you can be killed or, you know it can be massive. Plus it’s a risk to everybody else. So you must be healthy and happy.”* (FG5, metropolitan, blue collar, men and women)

In contrast to the beliefs reported above, it was reported by a handful of participants that there was little or no relationship between the health of employees and productivity and other desirable traits (e.g., cheerful demeanour and team player). A manager of a hotel mentioned:

*“Down where I work, some of the staff may be unhealthy, but whether or not I look at them as an unhealthy staff member, well that doesn’t really make much sense. Some of the healthiest staff there are the laziest staff there. And some of the people that are slow, oh not slow, but they don’t look healthy, they smash out work. They know what they’re doing and they get it done.”* (FG7, metropolitan, blue collar, men)

A few participants believed that workers could engage in high risk behaviours including regular illicit drug use, while still maintaining high levels of competence and success in their roles. This contradicts earlier descriptions of healthy workers.

*“Well I’d dispute that…I think the proof would be go to (the city), there would be many drug users, many lawyers on cocaine and are doing an exceptional job. So it depends what you’re talking about.”* (FG10, metropolitan, white collar, men)

### Employers’ beliefs around the role the workplace should play in influencing employees’ health and wellbeing

Generally, participants identified a need for healthy workers; however views varied between and within groups. Employers’ conceptualisation of workplace health and wellbeing also influenced their beliefs. Occupational health and safety was unanimously viewed as essentially the role of the workplace, however on the broad topic of health and wellbeing, views differed depending on industry and location and whether or not workplaces already prioritised health and wellbeing initiatives. Such comments were more pronounced in the rural areas and from participants working in blue collar industries.

*“During work hours I think yes. Because nowadays the liabilities are generally on the employer to push for safe work practice – was that a safe area that he was working in when he hurt himself? That would be more looked at than the actual person’s fatigue level or something like that.”* (FG1, rural, blue collar, men and women)

*“Obviously the health and safety of the workers is paramount at work.”* (FG3, rural, blue collar, men and women)

Safety aside, it was felt that employers were responsible for the health and wellbeing of employees, but that the level of responsibility and context varied. It was first and foremost up to the individual to maintain an acceptable level of personal health, while the workplace was often viewed as playing a more minor role that should support employee health. There was reluctance to enforce particular behaviours outside the scope of occupational health and safety as this was sometimes perceived to interfere with personal choice.

*Respondent 1: “Well certainly the employee is (responsible). The management can have a view and provide support, but I’ve been in organisations where they have a gym and people choose not to use it – it’s their choice. Or they have facilities for mental health which they don’t choose to use, so it’s really a case of providing the backup, but individuals are individuals and they do what they wish.”*

*Respondent 2: “Themselves first, and then management should be the people that are overlooking it and seeing what he’s doing wrong or if he’s eating bad or this and that, so it’s up to me it’s up to management to say, “Hang on mate, come here”. Talk to them on the side and say, “Try this. Try that. Have a problem come back to me”. That is what it is.”* (FG8, metropolitan, white collar, men)

As mentioned earlier, the responsibility of the workplace in terms of the mental health of their workers was a strong theme throughout the focus groups. This was also related to friendships in the workplace and the sense of responsibility, which was again, more salient in those from smaller businesses. There emerged a hierarchy of responsibility with safety at the top, followed by mental health, and then lifestyle factors at the bottom (e.g., nutrition and physical activity).

*“That is part of your job as a boss so as to keep an eye on the mental health of your workers to see where they’re actually travelling. You obviously don’t want to step into their personal lives but you’ve still got to be on the edge of it, see if there are problems with the family and that type of thing because that combined with fatigue is absolutely critical to your people.”* (FG1, rural, blue collar, men and women)

*“If we notice someone stressed we’ll pull them aside and have a chat to them, if we notice any, I don’t know, something unusual happening we’ll identify it if we can and have a chat to the staff to make sure there’s something not underlying that we can help with. And we have workplace counselling available where you can go and speak to somebody.”* (FG6, metropolitan, blue collar, women)

Around half of all participants reported having some sort of policy or program relating to mental health, cigarette use, alcohol consumption, healthy eating, or physical activity indicating some level of perceived responsibility beyond occupational health and safety issues. These included employee assistance programs, healthy staff menus, smoke free zones, provision of low and non-alcohol alternatives at work functions, and active transport policies. Such programs were rarely reported in small business settings.

In contrast to the findings reported above, some participants felt that the workplace did not play a role in influencing workers’ health and wellbeing. Such sentiments were often associated with two beliefs: (1) workplaces only exist to make money, and (2) the workplace should not attempt to control personal lifestyle behaviours. The latter belief was often articulated as the ‘nanny state’. The nanny state was discussed with anger and frustration by several participants especially those in the rural groups; one participant threw their paperwork into the middle of the table in a display of angry dismissal regarding the concept of creating health and wellbeing programs at work; thus highlighting the controversy of this topic for some participants.

*“If someone has got an unhealthy lifestyle, is the workplace the place to try and sort that out? I don’t think so. I think companies exist to make money they don’t exist to improve people’s lives. They come to work, work hard and you go home and if you’ve got issues you sort it out at home. I don’t think the workplace is a place to sort somebody’s issues out.”* (FG3, rural, blue collar, men and women)

*“And this is the grey area, if you’re talking about the organisation doing something for the individuals about their health, because normally that’s an individual’s choice, and most organisations wouldn’t see it as their responsibility. It’s a bit like a ‘nanny state’.”* (FG8, metropolitan, white collar, men and women)

*“I have a problem with the whole concept…our whole society is moving where individuals don’t take responsibility for themselves, and I’m not saying it’s wrong for a workplace to adopt this, I think it’s quite a healthy thing, but how much money do we spend on it?”* (FG4, rural, white collar, men and women)

To further explore the nanny state issues being raised, participants were asked to imagine that they could apply for funding to implement healthy workplace changes. Perhaps counterintuitively, *all* expressed a desire to implement changes if funding was provided. Their responses seemed to imply that many of their concerns regarding a nanny state were somewhat alleviated and indicated some sense of duty in relation to their employees’ health. Many of these responses were reported in an enthusiastic manner, with excitement expressed about the prospect of being able to help their colleagues to achieve healthier lifestyles. Such a finding highlights that many influences may be at play when employers are making decisions relating to the implementation of health promoting initiatives at work.

*Interviewer: “So what about if you could apply for a grant for up to $10,000 to improve the health and wellbeing of your workplace in line with the smoking, nutrition, alcohol, and physical activity areas. Would that be appealing?”*

*Respondent 1: “Yes. It would relieve our funding because we’re so tight on what we spend our money on. To know that you actually had that money to spend to promote wellbeing, hopefully that they will take on themselves at the end of the day – it would be great.”*

*Respondent 2: “Definitely. I think if it was any form of grant, I would turn around to my staff and buy a gym membership for everyone and say, “Look, you’re designated for two hours this week. You let me know what hours suit you to go the gym or whatever”, or some sort of a program.”*

*Respondent 1: “The benefit coming out of it that they would continue that physical activity or that healthy program and things like that. You can’t keep pouring it into it. You hope that from the initial push then people will try and keep that.”* (FG4, rural, white collar, men and women)

## Discussion

This phenomenological study explored some of the factors affecting employers’ views on the promotion of health and wellbeing in the workplace. The identified factors were complex and dynamic, with differences apparent according to the location, size, and industry of the workplaces represented in the study. The results from the present study provide insights that may assist researchers, health promoting organisations, practitioners, insurance companies, occupational physicians, various government departments, and labour unions to more effectively generate interest and action from employers to create improvements in employee health outcomes and associated work-focussed benefits. While the findings are most applicable to the Australian context, they may have relevance for international settings given the universal applicability of workplace health promotion and the growing trend towards the implementation of health and wellbeing programs in the workplace [13–19].

In the present study, workplace health appears entrenched within a health and safety paradigm among many of the employers (especially those from rural areas). It also appears to be more commonly understood in larger workplaces. In a recent research review, it was found that workplace health and wellbeing programs are less common in small compared to larger businesses [38]. This was largely the result of both direct program costs and indirect costs including staffing and time. Further, a qualitative study [39] involving 18 managers from small to medium sized businesses in the United Kingdom, found that there was limited awareness of what constitutes workplace health promotion. If representative of the wider organisational community, the promotion of workplace health and wellbeing is most likely to exist in larger businesses that have clearly distinguished it from their legal responsibility for health and safety. A workplace education campaign providing clear information about what constitutes health and wellbeing beyond the scope of occupational health and safety paradigms may be warranted to enable a dialogue within workplaces to open; this may be especially relevant for smaller workplaces.

The results of this study add to the field by providing definitions and descriptions of both healthy and unhealthy workers. Previous research demonstrates that employers’ beliefs and perceptions surrounding the importance of healthy workers may be key factors influencing the creation of workplace health and wellbeing initiatives [12]; while this was also found in the present study, the link was not nearly as convincing. More research that can further explicate this link and any moderating or mediating factors is required. Despite this, it is recommended that information on the potential benefits of the promotion of workplace health and wellbeing aligned with perceptions relating to healthy and unhealthy workers may be necessary to contextualise information provided.

The results of this study add to the existing literature demonstrating that while employers may be cognisant of the benefits of healthy workers, they remain uncertain about their personal or corporate responsibility to provide health promoting opportunities for their employees. Reluctance to direct employees on matters outside the scope of their job roles has been reported elsewhere [21, 24, 31, 38]. For example, McCoy et al. [38] found that in small businesses, employers expressed a strong disinclination to “meddle” in their employees’ lives in relation to the promotion of health and wellbeing initiatives. This reluctance may also reflect the views of some employers in this study that it is possible to be an effective employee while exhibiting poor lifestyle choices.

Employers from smaller workplaces in the current study were more likely to describe feeling personally responsible for their employees’ health, particularly their mental health. This contrasts with employers from larger workplaces who were less likely to express that it was appropriate for them to make suggestions relating to their employees’ lifestyle choices. Workplaces that have a high risk of injury, such as mining, were focused on reducing injury risk, which may include strategies to reduce alcohol and other drug consumption, treat mental health issues, and improve the physical fitness of employees. However, such strategies are within the context of reducing immediate threats to injury, rather than influencing the broader lifestyle choices of employees. In these workplaces, once the decision to change work practices is made, employers are empowered to implement new policies and practices. This contrasts with other similar sized businesses in different industries, or medium and small businesses in other industries, where the risk of serious injury at work was considered unlikely and therefore employers expressed greater reluctance to suggest or dictate changes in work practices for health purposes. Previous research has shown that employers typically do not feel responsible for influencing their employees’ health behaviours [21–23], unless there is a risk of injury. Moreover, prior research demonstrates that the way in which ‘responsibility’ is perceived between employers and employees is different, whereby employers and other stakeholders (e.g., labour unions and insurance companies), view responsibility as being akin to duty [21]. In contrast, employees view responsibility as the equivalent to autonomy. It has been suggested that this conceptual incompatibility may result in distrust between stakeholder groups [21]. Such a finding may have important implications, in light of the results from the present study, especially as they relate to the need for clear communication regarding different stakeholders’ positions on responsibility (e.g., nanny state concerns).

While many participants in the present study did not report feeling *responsible* for their employees’ health, they expressed *concern* for their employees’ mental and physical health because they had established personal friendships (as per Linnan et al. [22]). This seemed to be a very salient theme for employers from smaller businesses where participants interacted directly with their employees and developed close personal relationships. Participants from larger businesses did not mention friendship; this may be the product of a corporate culture that physically and corporately separates owners, managers, and workers. While employers from larger businesses may not be working directly with many of their employees, friendships do exist among team members that resemble small business employer attitudes towards their staff. It is possible that within larger organisations opportunities may exist for team leaders and supervisors to positively influence their group members’ health behaviours (as per Harter et al. [40]), although this requires further investigation.

Two integral components of a healthy workplace are the health of employees and the performance of workplaces [41]. Productivity is linked with profitability and when workplace health promotion is presented as a productivity measure, employers view it as a financial return on investment decision [9]. However, there is at best modest and not well quantified evidence that implementing a health promotion initiative in the workplace increases productivity or profitability [9, 17]. In this study, when the promotion of workplace health and wellbeing was presented to employers as a way to positively influence their employees’ lives, and financial considerations were put to one side, those who had previously expressed nanny state concerns unexpectedly became enthusiastic about the introduction of health-promoting initiatives. With financial barriers removed, participants did not consider the financial return on investment and shifted their focus to generating a range of workplace health strategies and how they might be implemented, rather than focusing on the productivity justification for introducing these types of initiatives. Given the wide range of workplace health promotion strategies that can be introduced with little or no cost, further investigation into the potential for moving away from a return to investment approach is warranted. This may lead to gains in workplace health promotion uptake.

Finally, the morale of the workforce was an important perceived benefit associated with healthy workers, they were more likely to consider their implementation if they believed these types of initiatives would improve the health or morale of their employees and whether the company could afford the implementation costs. These findings support efforts to be clear about the benefit of each strategy as well as the non-financial rewards of implementing strategies.

### Strengths and limitations

As the findings presented are qualitative in nature, it is not possible to generalise the information provided beyond the scope of the sample participating in the research. In particular, the views expressed may not be representative of employers from other areas within the country or internationally. However, the data generated provide important information that can assist health promoting organisations, researchers, practitioners, insurance companies, occupational physicians, various government departments, and labour unions to more effectively generate interest and action from employers. Future research could be directed towards testing and quantifying these themes so as to advance an understanding of the pathway to successful workplace health and wellbeing initiatives, programs, and policies. This would help to improve the capacity of workplaces wanting to effectively implement healthy changes and generate information that more clearly explicates the drivers of this type of change in the workplace.

## Conclusions

The results of this study indicate that several factors influence employers’ views on the appropriateness of workplace health and wellbeing initiatives and it is important to understand how best to target employers from different business sizes, industries and geographical locations. When it comes to influencing employees’ health and wellbeing beyond the scope of occupational health and safety, a multi-faceted approach involving education about what workplace health and wellbeing encapsulates is warranted. Further, information on the potential benefits of the promotion of workplace health and wellbeing aligned with perceptions relating to healthy and unhealthy workers may be necessary to contextualise information provided. Finally, different workplace circumstances must be given consideration when designing initiatives and interventions.
